# Development and application of a deep learning-based comprehensive early diagnostic model for chronic obstructive pulmonary disease

**DOI:** 10.1186/s12931-024-02793-3

**Published:** 2024-04-18

**Authors:** Zecheng Zhu, Shunjin Zhao, Jiahui Li, Yuting Wang, Luopiao Xu, Yubing Jia, Zihan Li, Wenyuan Li, Gang Chen, Xifeng Wu

**Affiliations:** 1 Center of Clinical Big Data and Analytics of The Second Affiliated Hospital and Department of Big Data in Health Science School of Public Health, Zhejiang University School of Medicine, Hangzhou, Zhejiang China; 2 Department of Respiratory and Critical Care Medicine, The Second Affiliated Hospital of Zhejiang University School of Medicine, Lanxi Branch (Lanxi People’s Hospital), Hangzhou, Zhejiang China; 3 College of Computer Science and Technology, Zhejiang University, Hangzhou, Zhejiang China; 4 National Institute for Data Science in Health and Medicine, Zhejiang University, Hangzhou, Zhejiang China; 5 The Key Laboratory of Intelligent Preventive Medicine of Zhejiang Province, Hangzhou, Zhejiang China

**Keywords:** COPD, Deep learning, Diagnostic, Data fusion

## Abstract

**Background:**

Chronic obstructive pulmonary disease (COPD) is a frequently diagnosed yet treatable condition, provided it is identified early and managed effectively. This study aims to develop an advanced COPD diagnostic model by integrating deep learning and radiomics features.

**Methods:**

We utilized a dataset comprising CT images from 2,983 participants, of which 2,317 participants also provided epidemiological data through questionnaires. Deep learning features were extracted using a Variational Autoencoder, and radiomics features were obtained using the PyRadiomics package. Multi-Layer Perceptrons were used to construct models based on deep learning and radiomics features independently, as well as a fusion model integrating both. Subsequently, epidemiological questionnaire data were incorporated to establish a more comprehensive model. The diagnostic performance of standalone models, the fusion model and the comprehensive model was evaluated and compared using metrics including accuracy, precision, recall, F1-score, Brier score, receiver operating characteristic curves, and area under the curve (AUC).

**Results:**

The fusion model exhibited outstanding performance with an AUC of 0.952, surpassing the standalone models based solely on deep learning features (AUC = 0.844) or radiomics features (AUC = 0.944). Notably, the comprehensive model, incorporating deep learning features, radiomics features, and questionnaire variables demonstrated the highest diagnostic performance among all models, yielding an AUC of 0.971.

**Conclusion:**

We developed and implemented a data fusion strategy to construct a state-of-the-art COPD diagnostic model integrating deep learning features, radiomics features, and questionnaire variables. Our data fusion strategy proved effective, and the model can be easily deployed in clinical settings.

**Trial registration:**

Not applicable. This study is NOT a clinical trial, it does not report the results of a health care intervention on human participants.

## Introduction

Chronic obstructive pulmonary disease (COPD), a prevalent and preventable chronic lung ailment, is a well-established risk factor for lung cancer [1–3]. In 2019, COPD resulted in approximately 3.23 million global deaths [4], with a reported prevalence of 13.7% in individuals aged 40 and above in China [5]. The insidious onset, atypical symptoms, and limited public awareness contribute to a low success rate in early diagnosis and a high case-fatality rate [6, 7]. Strategies to enhance early COPD diagnosis are therefore in urgent demand [8, 9].

Conventional diagnostic methods, primarily relying on pulmonary function tests, exhibit limitations in accuracy, demonstrating a sensitivity of 79.9% (74.2–84.7%) and specificity of 84.4% (68.9–93.0%) [10] due to the subtle clinical symptoms and modest pulmonary function changes in early-stage COPD [10, 11]. In contrast, Computed Tomography (CT) imaging emerges as a more objective, precise, and efficient tool for the early diagnosis and assessment of COPD [12, 13], and improves sensitivity to 83.95% (73.0–89.0%) and specificity to 87.95% (70.0-95.6%) [14]. However, the disease’s heterogeneity poses practical challenges for conventional manual reading methods, including subjective interpretation variability among medical personnel and time-consuming processes [12–15].

To address these challenges, artificial intelligence (AI) techniques, particularly machine learning, have been applied in the analysis of CT-based radiomics features and demonstrated favorable performance [16]. In recent years, deep learning, a subset of machine learning known for its ability to handle complex problems, has further expanded the application of AI across various fields, including medicine [17, 18]. Within the context of COPD, deep learning algorithms such as convolutional neural networks (CNNs) have significantly improved diagnostic performance [19–25]. Notably, an AUC of 0.82 was achieved for COPD detection using DenseNet [26], a robust deep learning network. Additionally, leveraging radiomics features extracted from deep learning models to predict survival for COPD patients resulted in a concordance index of 0.73 (95% CI, 0.70–0.73) [27]. Furthermore, some recent studies have utilized deep learning approaches to model respiratory sound for COPD diagnosis, achieving an accuracy of 0.953 [28], an AUC of 0.966 [29] and an accuracy of 0.958 [30], respectively. These deep learning models were able to autonomously extract intricate features from CT images, and provide exceptional precision in image analysis and alleviate the workload of clinical practitioners [18–20, 26].

Fusion strategies were recently introduced where radiomics features and abstract deep learning features were simultaneously extracted from CT images, and computational algorithms such as Multi-Layer Perceptron (MLP) [31] were then employed to seamlessly integrate these cross-modal features, leading to the construction of fusion models [32]. Leveraging the complementary nature of radiomics and deep learning features, this fusion approach holds promise in enhancing precision and reliability in diagnosis and risk prediction [32]. Moreover, as efforts continue in constructing large population cohorts, questionnaire data are becoming more widely available. Existing research has demonstrated that adding radiomic features to epidemiological information can improve the predictive capacities of machine learning models for COPD progression and mortality [33, 34].However, whether these questionnaire data can enhance the diagnostic performance of deep-learning based models of COPD remains unclear.

In this study, we combined deep learning with radiomics profiles from CT images using MLP to build a COPD diagnostic fusion model. The epidemiological questionnaire data were then incorporated to develop a comprehensive model. A comparative analysis was conducted to assess the performance of standalone, fusion, and comprehensive models.

## Materials and methods

### Study design and COPD ascertainment

We obtained a CT dataset from Lanxi People’s Hospital, comprising 23,552 chest CT scans from 12,328 adults, with each adult contributing a varying number of scans. A team of respiratory specialists at the hospital reviewed CT diagnostic reports and related symptoms, and confirmed the presence of normal lung radiographic findings or imaging manifestations indicative of COPD in 2,983 adults. Specifically, normal lung radiographic findings were defined as the absence of obvious pathological lesions in both lungs, as indicated in the imaging reports. Imaging manifestations of COPD were characterized by the presence of chronic bronchitis combined with emphysema or bullae, or a definitive diagnosis of COPD. These manifestations could also be accompanied by pulmonary nodules, calcified lesions, fibrotic changes, minor inflammation (all less than 1 cm), or minor pleural effusion (less than 3 cm), as well as localized bronchial dilation. CT imaging reports showing other pathological abnormalities, as well as asthma patients, were excluded from the study. Furthermore, 2,317 of these 2,983 individuals had responded to a comprehensive epidemiological questionnaire as part of the Healthy Zhejiang One Million People Cohort, a newly established prospective cohort in Zhejiang province, China. No patients were excluded based on gender or ethnicity.Although pulmonary function tests (PFTs) are commonly recommended for the diagnosis of COPD, they possess certain limitations in early detection of COPD, and some patients with severe symptoms may be unable to tolerate or fully complete these tests, resulting in incomplete PFT results in our daily practice. Using clinicians’ actual diagnoses as the criteria aligns with our goal of creating a reliable, non-invasive, and practical diagnostic tool.

### Questionnaire data acquisition

The epidemiological questionnaire gathered data on participants’ demographic variables, lifestyles, and health status. All questions were asked and answered in Chinese.

Demographic variables included age, gender, residence (urban or rural), marital status (married, divorced/separated, widowed, not married, or others), years of education (0, ≥ 1 and ≤ 6, ≥7 and ≤ 12, or ≥ 13 years), annual income rounded in thousands (< 50, 50–100, 101–200, 201–300, or > 300, thousand Chinese Yuan), and number of cohabitants in the household (< 2, 2, 3, 4, > 4).

Body Mass Index (BMI) was calculated by dividing weight (in kilograms) by the square of height (in meters). Subsequently, individuals were categorized into different BMI groups, including underweight (< 18.5 kg/m^2^), healthy weight (18.5 ≤ BMI < 24 kg/m^2^), overweight (24 ≤ BMI < 28 kg/m^2^), and obesity (≥ 28 kg/m^2^) categories.

Smoking status was classified based on participants’ self-reported smoking history in the questionnaire. Specifically, participants who reported smoking fewer than 100 cigarettes in their lifetime were classified as “never smokers”. Those who reported smoking more than 100 cigarettes in their lifetime and had quit smoking at the time of the questionnaire survey were classified as “former smokers”. Lastly, participants who reported smoking more than 100 cigarettes in their lifetime and were still smoking at the time of the survey were classified as “current smokers”. Smoking addictiveness was categorized as none, moderately addicted or highly addicted based on the Fagerstrom Test for Nicotine Dependence (FTND). Participants also provided information on the weekly frequency of secondhand smoke exposure, categorized as never, sometimes, 1–2 days/week, 3–5 days/week, or almost daily. Drinking status was categorized as never, former drinker or less than once per week, more than once per week for less than 12 years, or more than once per week for 12 or more years.

Diet preferences, habits, and frequency of behaviors were also collected. Preferences included food temperature, dryness, texture, saltiness, spiciness. Habits included speed of eating, meal regularity, and balance of diet (rich in meat, rich in vegetables, or balanced). Frequencies of dietary behaviors per week included intake of red meat, vegetables, whole grains, oil-rich food, sugar-rich food, sugar-sweetened beverages, tea, and leftovers, along with the frequency of dining out, and breakfast. Participants also disclosed whether they were vegan.

Weekly work intensity was classified based on the work duration (< 20 h, 20–40 h, currently working and ≥ 40 h, retired and ≥ 40 h).

Participants were asked to report the intensity, frequency, and duration of physical activity that they did during the past weeks, and then divided as inactive (0 metabolic equivalent (MET) hours /week), > 0 and < 7.5 MET hours/week, ≥ 7.5 and < 15.0 MET hours/week, and ≥ 15.0 MET hours/week.

Sleep quality was assessed based on items from the Pittsburgh sleep quality index (PSQI); depressive symptoms was evaluated based on the Center for Epidemiological Studies Depression-10 (CES-D-10) scale; and cognitive function was assessed based on the AD8 scale.

Health status data including prevalence of tumors, coronary heart disease, stroke, diabetes mellitus, osteoarthritis, Helicobacter pylori infection, gastritis, uremia, rheumatoid arthritis, and alcoholic fatty liver were also collected.

In modelling phase, Z-score normalization was applied to numerical questionnaire variables, while categorical questionnaire variables were encoded manually as binary (0 and 1) or ordinal values.

### Chest CT Image Acquisition and Preprocessing

This study utilized two CT scanners, the GE Optima CT680Q and the GE Optima CT540, for CT scanning. The scans were performed in a supine position after complete inspiration, from the lung apex to the lung base. Scanning parameters: tube voltage 120KV, adaptive tube currents according to the body size (ranging from 60 to 350 mA), detector configuration 64 × 0.6 mm, gantry rotation time 0.5s/rotation, pitch 1.0, matrix 512 × 512. The CT scanning had an attenuation coefficient range of -1024 to 3072 HU. Slice thickness was 1.25 mm. The scanned CT data were stored in DICOM format. For image preprocessing, we initially normalized the pixel density values of all CT scans to a range between 0 and 1. Subsequently, we employed the LungMask tool [35], which is specifically designed for automated lung segmentation in chest CT scans, and can be used as a python module, to obtain the lung parenchyma, with pixel values outside this region being set to zero. Its robust performance has been validated across diverse datasets and disease contexts. Finally, the resulting images were resized to a dimension of 128 × 256 × 256.

### Model development and evaluation

We propose a two-stage multimodal prediction modelling framework, wherein the first stage focused on feature extraction from various modalities. Specifically, epidemiological features were derived from preprocessed raw questionnaire data, while deep learning features were extracted from preprocessed CT images using self-supervised learning generative model, and radiological features were obtained via lung image analysis tools. In the second stage, multimodal features were fused. Initially, features from different modalities were dimensionally reduced to preset dimensions using different shaped multilayer perceptron (MLP). Subsequently, these features were concatenated to construct multimodal feature utilized for prediction. Finally, the multimodal features were fed into the stacked MLP to output the final prediction.

#### Radiomics feature extraction

The radiomic features were extracted from the resultant CT images using the PyRadiomics [36] package. Each CT image was resampled to (1,1,1) before feature extraction. The extraction parameters were configured as follows: ‘minimumROIDimensions’ set to 2, ‘minimumROISize’ left as None, ‘normalize’ set to False, ‘normalizeScale’ set to 1, ‘removeOutliers’ left as None, ‘resampledPixelSpacing’ set to (1,1,1), ‘interpolator’ specified as ‘sitkBSpline’, ‘preCrop’ set to False, ‘padDistance’ set to 5, ‘distances’ configured as (1), ‘force2D’ set to False, ‘force2Ddimension’ set to 0, ‘resegmentRange’ left as None, ‘label’ assigned as 1, and ‘additionalInfo’ left as True.

In addition to the original image, we applied a range of filters including the Wavelet, Laplacian of Gaussian (LoG), Square, Square Root, Logarithm, Exponential, Gradient, and Local Binary Pattern (LBP) filters to the images. Features were extracted using First order, Gray Level Cooccurrence Matrix (GLCM), Gray Level Size Zone Matrix (GLSZM), Gray Level Run Length Matrix (GLRLM), Gray Level Dependence Matrix (GLDM), Neighboring Gray Tone Difference Matrix (NGTDM), and Shape methods. A total of 2,983 participants had extractable CT imaging radiomics features using the PyRadiomics package, with a consistent yield of 1,409 radiomics features for each subject. In constructing the model, Z-score normalization was applied.

#### Self-supervised deep learning feature extraction

We initially utilized Variational Autoencoder (VAE) [37], a large-scale unlabeled self-supervised model to extract deep learning features with dimensions of 16 × 32 × 32 from the 23,552 images. Among them, we subsequently employed 3D convolutional kernels to down-sample the deep learning features in 2,983 CT images containing available radiomics features. The down-sampling was achieved using 3D convolutional kernels with a kernel size of (3, 3, 3) and a stride of (1, 1, 1), while maintaining the same number of input and output channels. After passing through this convolutional kernel, the output feature map’s dimensions are halved compared to the input feature map. This down-sampling resulted in deep learning features with dimensions of 8 × 16 × 16. Finally, we flattened these reduced-dimension features, yielding 2,048 deep learning features for each participant.

The optimization objective during the training of the unlabeled 3D VAE aimed to maximize the Evidence Lower Bound (ELBO), which consists of two components: the reconstruction loss and the prior matching loss. Additionally, to ensure the effectiveness of the feature representations, we incorporated patch adversarial loss and perceptual loss to enhance the quality of image reconstructions. These added components were instrumental in preserving the quality of the reconstructed images while optimizing the feature variables.

#### Feature fusion strategy

We employed the MLP model for feature fusion. The fusion strategy was feature-specific, meaning a uniform fusion approach was applied to participants concurrently exhibiting different features. Therefore, we utilized MLP models in two subsets: one comprising 2,983 participants with both radiomics and deep learning features for the fusion model, and another with 2,317 participants possessing radiomics, deep learning, and epidemiological features for the comprehensive model.

To reduce dimensionality, we employed a down-sampling process that reduced the deep learning features from 2,048 to 256 dimensions and the radiomic features from 1,409 to 139 dimensions. Here the down-sampling process was achieved by setting the output dimension of the MLP, which was a hyperparameter. However, the original dimension of 49 epidemiological features remained unchanged.

**Fig. 1:**
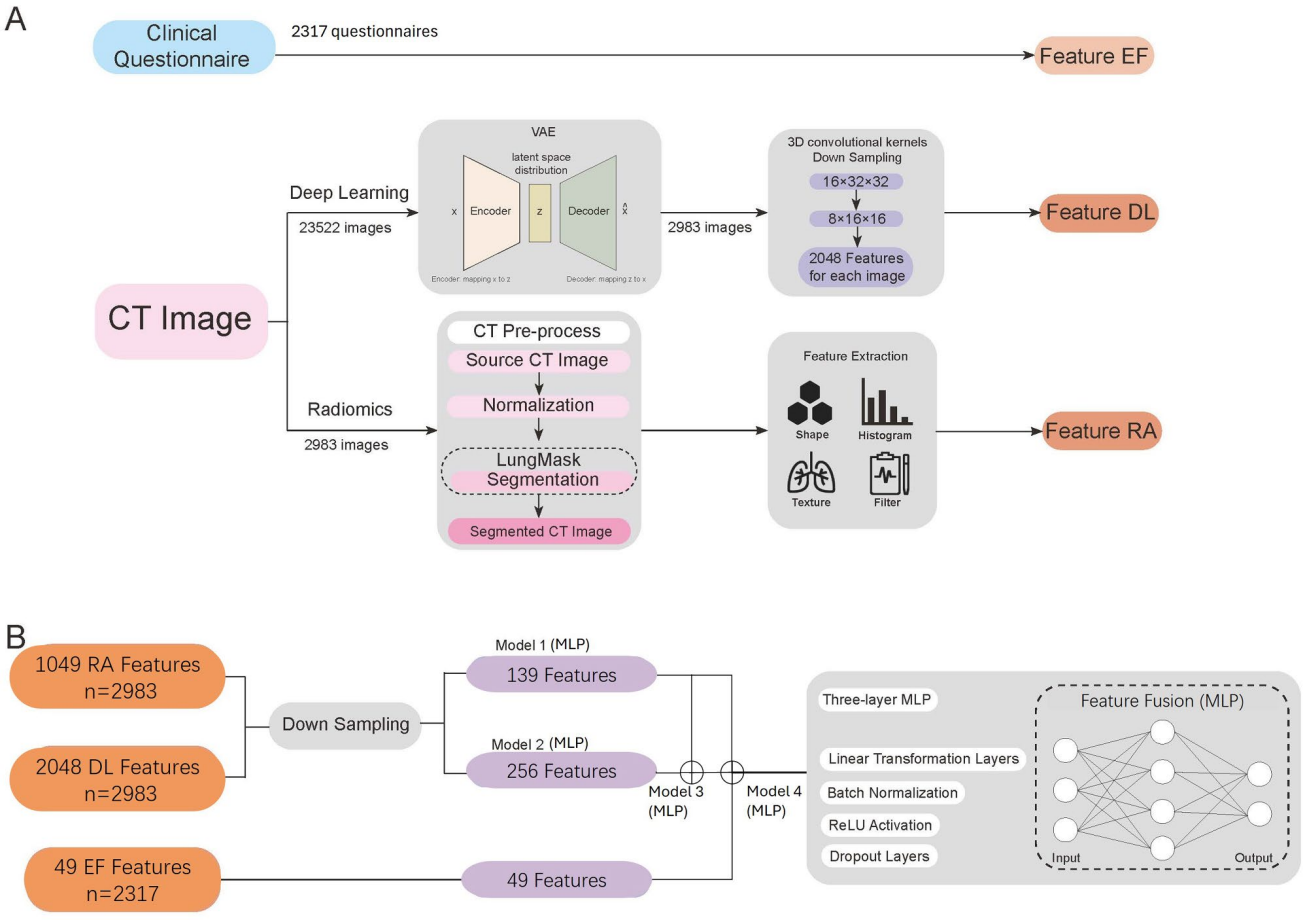
Flowchart of standalone, fusion, and comprehensive models. Model 1: standalone model based on radiomics features; Model 2: standalone model based on deep learning features; Model 3: fusion model; Model 4: comprehensive model. Note: VAE: Variational Autoencoder; Feature EF: epidemiological features; Feature DL: deep learning features; Feature RA: radiomics features; MLP: Multi-Layer Perceptron

These features were subsequently concatenated and processed through a three-layer MLP module. Each module within the MLP architecture was composed of linear transformation layers, batch normalization, rectified linear unit (ReLU) activation, and dropout layers. Figure 1 shows the workflow of our fusion strategy.

#### Hyperparameters selection

##### VAE

Our VAE model was trained with a carefully chosen set of hyperparameters to optimize performance. For model construction, we set the number of groups for group normalization to 32, and allocated 64, 128, and 128 feature channels per layer within the encoder. In the loss function, we assigned coefficients of 1e-3, 1e-2, and 1e-6 to the perceived loss, confrontation loss, and Kullback-Leibler loss, respectively. We utilized the Adaptive Moment Estimation (Adam) optimizer with an initial learning rate of 1e-4 for generator and 5e-4 for discriminator. Finally, training was conducted over 100 epochs to ensure comprehensive model training and convergence.

##### MLP

Our MLP model underwent training with a set of hyperparameters to optimize performance. We employed the Stochastic Gradient Descent (SGD) optimizer with an initial learning rate of 0.01 and momentum of 0.9. The learning rate was decayed every 30 epochs by a factor of 0.3 to facilitate convergence. To prevent overfitting, L2 weight decay with a coefficient of 0.01 was applied. A batch size of 200 and a hidden layer width of 64 were chosen for enhanced computational efficiency and model capacity. Furthermore, a dropout ratio of 0.5 was introduced to mitigate overfitting. Finally, training was conducted over 160 epochs to ensure comprehensive model training and convergence.

#### Time complexity analysis

We utilized the big O notation to show the time complexity of models above. The time complexity of the convolution module is

$$\begin{aligned}&Time \sim O ({\sum }_{l=1}^{L}\left({G}_{l}\cdot{{C}_{l-1}\cdot F}_{h}\cdot{F}_{w}\cdot{F}_{d}\right)\\&+\left({F}_{h}\cdot{F}_{w}\cdot{F}_{d} \right)\cdot {K}_{l}^{3}\cdot {C}_{l}\cdot{C}_{l-1})\end{aligned}$$

Where $G$ represents the number of groups in group normalization, ${F}_{h},{F}_{w},{F}_{c},C$ represents the length, width, height and number of channels of the feature map, $K$ represents the size of the convolutional kernel and $L$ represents the number of convolutional kernel modules; The time complexity of the down sampling module is

$$Time \sim O({\sum }_{l=1}^{{ \text{L}}_{\text{d}}}\left({F}_{h}\cdot{F}_{w} \cdot {F}_{d} \right) \cdot {K}_{l}^{3} \cdot {C}_{l} \cdot {C}_{l-1}) )$$)

The time complexity of the self-attention module is.

$$Time \sim O({N}^{2}+Nd$$)

Where N represents the sequence length and D represents the feature dimension.

### Other statistical methods

Continuous variables were presented as mean ± SD and categorical variables were presented as count and percentages. Model performance was evaluated using metrics such as accuracy, precision, sensitivity, F1-score, Brier score, receiver operating characteristic curve (ROC), and area under the curve (AUC). All metrics were obtained using weighted methods except for the Brier score. Additionally, bootstrap resampling was used to compute the 95% confidence intervals for accuracy, precision, sensitivity, and F1-score, with 1000 randomized tests. Accuracy indicates overall correctness, sensitivity measures true positive rate, precision assesses positive prediction accuracy, and F1-score balances both precision and sensitivity through harmonic mean [38]. These metrics are prevalent in classification performance evaluation [39–42], where higher values of AUC denote model’s superior discrimination ability between positive and negative classes. A two-tailed P-value of less than 0.05 indicated statistical significance. Statistical analysis was conducted using Scipy packages in Python 3.9. The CT images were processed with SimpleITK for preprocessing and Lungmask for radiomics feature extraction. Self-supervised 3D- VAE and MLP models were implemented in Python 3.9 using Pytorch 1.13.1.

## Results

### Patients characteristics

The study included a total of 2,983 participants, which comprised 497 COPD patients and 2,486 participants with normal lung radiographic findings. Questionnaire data were available for 2,317 participants, where 477 were COPD patients.

To improve the comparability of models, the 2,317 participants with available questionnaire variables were randomly divided into training and testing datasets at an 8:2 ratio. The training set encompassed 1,853 participants, including 373 individuals with COPD and 1,480 individuals without COPD; and the testing set comprised 464 individuals, with 104 having COPD and 360 without COPD. Then, for the additional 666 individuals in the total 2,983 individuals, a same division strategy was applied to establish another training and testing sets. As a result, the final training set included 2,385 individuals, comprising 389 with COPD and 1,996 without COPD; and the final testing set comprised 598 participants, with 108 having COPD and 490 without COPD. Table 1 provided a detailed overview of the basic epidemiological characteristics of both the training and testing sets. Importantly, statistical analysis revealed no significant differences between them.

**Table 1:** The basic characteristics of training and test sets of the 2,317 participants

Characteristics	Train set	Test set
Age, years	62.1 ± 12.6	61.9 ± 12.4
BMI, kg/m^2^		
BMI < 18.5	126(6.8%)	37(8.0%)
18.5 ≤ BMI < 24	1009(54.4%)	248(53.4%)
24 ≤ BMI < 28	561(30.3%)	143(30.8%)
BMI ≥ 28	157(8.5%)	36(7.8%)
Gender		
Male	876(47.3%)	238(51.3%)
Female	977(52.7%)	226(48.7%)
Residence		
Rural	1521(82.1%)	370(79.7%)
Urban	332(17.9%)	94(20.3%)
Education years, years		
0	496(26.8%)	107(23.1%)
1–6	616(33.2%)	161(34.7%)
7–12	650(35.1%)	176(37.9%)
≥ 13	91(4.9%)	20(4.3%)
Annual income in thousands, Chinese Yuan		
< 50	645 (40.1%)	162(40.2%)
50–100	646(40.2%)	175(43.4%)
110–200	276(17.2%)	57(14.1%)
210–300	25(1.6%)	6(1.5%)
> 300	16(0.9%)	3(0.7%)
Marriage Status		
Married	1630(88.0%)	398 (85.8%)
Divorced/Widowed	163(8.8%)	44(9.5%)
Never Married	60 (3.2%)	22(4.7%)
Smoke Status		
Current smoker	279(15.1%)	79(17.0%)
Former smoker	123(6.6%)	36(7.8%)
Non-smoker	1451(78.3%)	349(75.2%)
Second-hand smoke exposure		
Never	1290(70.3%)	321(69.3%)
Sometimes	308(16.8%)	72(15.6%)
1–2 days/week	33(1.8%)	3(0.6%)
3–5 days/week	51(2.8%)	16(3.5%)
Almost every day	152(8.3%)	51(11.0%)
Drinking status		
Never	1316(71.0%)	326(70.3%)
Sometimes	157(8.5%)	41(8.8%)
< 12 years	29(1.6%)	9(1.9%)
≥ 12 years	351(18.9%)	88(19.0%)
COPD		
COPD	373(20.1%)	104(22.4%)
Non-COPD	1480(79.9%)	360(77.6%)

*Note*: Data are mean ± SD or N(%), COPD: chronic obstructive pulmonary disease, CHD: coronary heart disease

### Diagnostic performance

We conducted a thorough assessment of the diagnostic performance of both standalone and fusion models. For COPD diagnosis, the AUC was 0.844 for the model utilizing deep learning features, and 0.944 for the model employing radiomics features. Notably, the fusion model outperformed the independent models, achieving an AUC of 0.952. In the comprehensive model, which incorporated deep learning, radiomics and epidemiological features, the performance was further enhanced, yielding an AUC of 0.971. Furthermore, the comprehensive model outperformed all other metrics, including accuracy, precision, recall, F1-score, and Brier score, which were separately 0.886, 0.909, 0.886, 0.891 and 0.080.

**Fig. 2:**
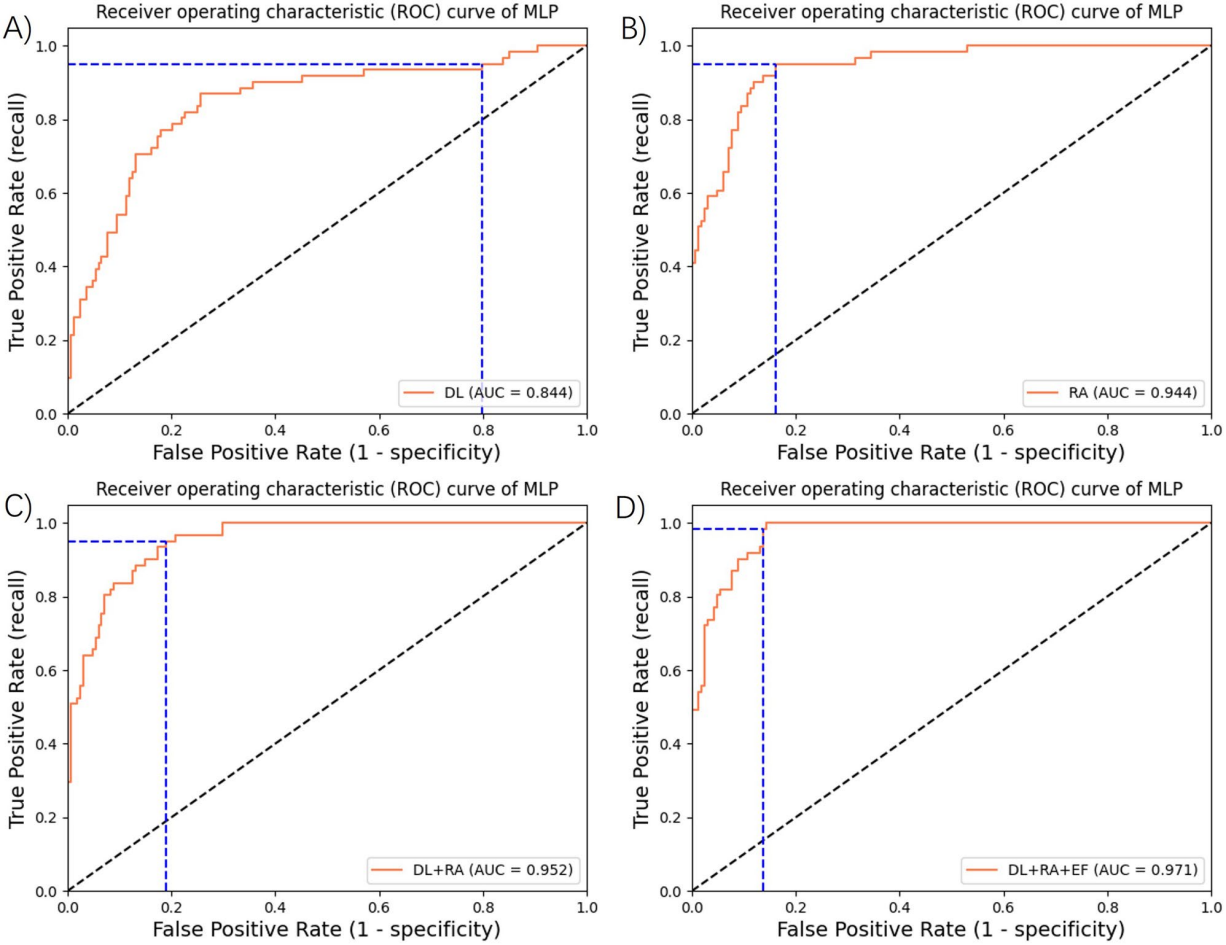
The ROC curves and their corresponding AUC values of various COPD diagnostic models. (**A**) standalone model based on deep learning features. (**B**) standalone model based on radiomics features. (**C**) fusion model integrating deep learning and radiomics features. (**D**) comprehensive model incorporating deep learning, radiomics and epidemiological features. RA: Radiomic Feature, DL: Deep Learning Feature, EF: Epidemiological Features

Figure 2 was a visual representation of the ROC curves and their corresponding AUC values for various models under consideration. Higher AUC value indicated a superior discriminatory capacity of a model.

Table 2 provided a comprehensive summary of the diagnostic metrics, including AUC, accuracy, precision, recall, F1 score and Brier score, for the different models assessed in the testing set. Our observations consistently indicated that among the two independent models, the radiomics-based model exhibited superior performance.

**Table 2:** Performance of various models for COPD diagnosis in test set

Models(n)	AUC	Accuracy	Precision	Recall	F1-score	Brier score
DL (*n* = 2,983)	0.844	0.782(0.747,0.782)	0.822(0.822,0.829)	0.782(0.747,0.782)	0.792(0.771,0.792)	0.154
RA (*n* = 2,983)	0.944	0.882(0.882,0.908)	0.882(0.882,0.906)	0.882(0.882,0.908)	0.882(0.882,0.907)	0.085
DL + RA (*n* = 2,983)	0.952	0.869(0.869,0.886)	0.887(0.887,0.909)	0.869(0.869,0.886)	0.873(0.873,0.893)	0.091
DL + RA + EF (*n* = 2,317)	0.971	0.886(0.856,0.886)	0.909(0.900,0.909)	0.886(0.856,0.886)	0.891(0.867,0.891)	0.080

RA: Radiomic Feature, DL: Deep Learning Feature, EF: Epidemiological Feature

Across all metrics, the fusion model consistently outperformed the independent models, reaffirming its effectiveness in enhancing diagnostic accuracy. Notably, the most remarkable performance was achieved by the comprehensive model.

### Model interpretability

**Fig. 3:**
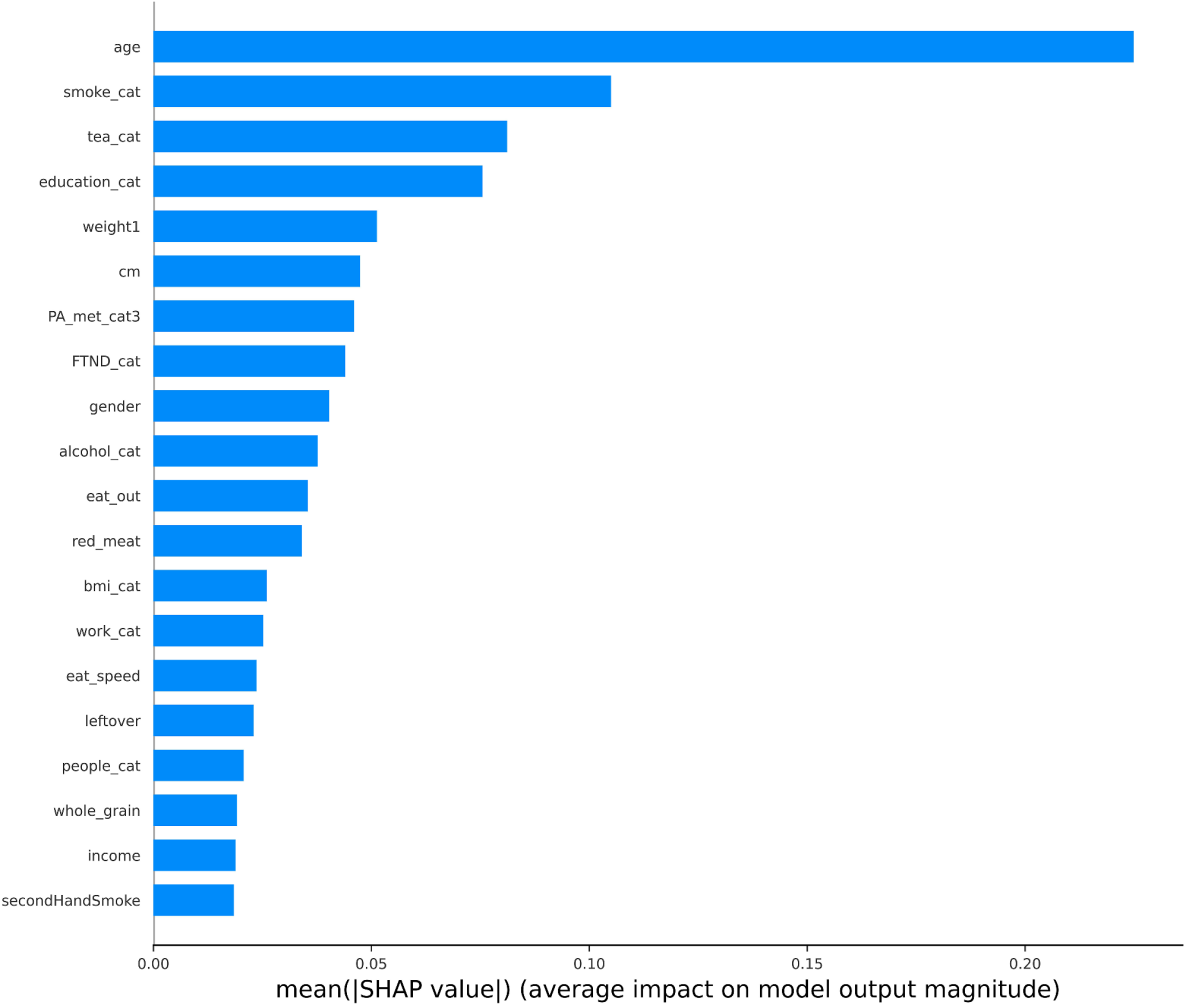
The SHAP values of epidemiological variables in the comprehensive model

We employed SHAP values to evaluate the relative importance of the epidemiologic variables in our comprehensive model, and the results were illustrated in Fig. 3. The SHAP values represented the average impact of each variable on the magnitude of the model output, with higher SHAP values indicating a more substantial role played by the respective variable.

## Discussion

We established and validated a robust AI-driven data fusion-based COPD diagnostic model. Leveraging deep learning features, radiomics, epidemiological data, and MLP, our fusion model achieved an AUC of 0.971 for diagnosis of COPD. Moreover, our modelling framework can be readily deployed in assisting COPD diagnosis in clinical practices. To the best of our knowledge, we are among the first studies that successfully applied a multimodal data-fusion strategy in COPD diagnosis in a Chinese population.

In recent years, large population cohorts such as COPDGene [43], ECLIPSE [44], and SPIROMICS [45] have been established in western countries, and these initiatives have significantly advanced the research on COPD. However, it is essential to acknowledge certain drawbacks, including the need for robust validation and potential challenges in generalizing findings to diverse populations. Notably, there remains a substantial gap in research related to COPD in Chinese population. The COMPASS (Investigation of the Clinical, Radiological and Biological Factors, Humanistic and Healthcare Utilisation Burden Associated with Disease Progression, Phenotypes and Endotypes of COPD in China) is a prospective multi-center study started in 2019, which has marked a recent effort in understanding the relevance of COPD biomarkers identified in Western cohorts to Chinese patients [46]. Our study bears unique significance as it employed an extensive dataset of CT scans from individuals residing in the eastern region of China, presenting a COPD diagnostic model tailored specifically for the Chinese population.

Owing to COPD’s slow progression and complex pathophysiology [47, 48], traditional lung function indicators like FEV1 [49, 50] have proven inadequate for early detection of COPD. For example, in a follow-up study, it was reported that approximately half of the 332 individuals with COPD at the end of the observation period exhibited normal FEV1 values by the age of 40 (*P* < 0.001) [49]. Conversely, CT scans offer the advantage of detecting structural lung changes at an earlier stage [51]. Previous studies have attempted to utilize mathematical models [52, 53] to assess emphysema, which largely represents lung parenchymal destruction [54]. In a study using integrated CT metrics, radiomics, and standard spirometry measurements, the investigators were able to estimate the presence and severity of emphysema at AUCs of 0.86 and 0.88, respectively [53]. However, these methods relied on manual annotation, which led to time consumption and issues related to observer variability [32, 48, 55]. The advent of deep learning has revolutionized the data extraction process and alleviated observer variability concerns [56]. Studies that employed deep learning algorithms with CT data for COPD detection had achieved notable success with AUCs of 0.87, 0.90 [57], and 0.927 [58], which were relatively good. However, previous studies have predominantly focused on radiomics features. For instance, one study aimed to predict COPD progression by combining radiomic features and demographics with a machine learning approach, achieving an AUC of 0.73 [59]. Another study extracted radiomics features for COPD survival prediction using a deep learning approach, resulting in a concordance index of 0.716 [27]. While the results were promising, whether adapting a multimodal feature fusion strategy would enhance the model performance remains unclear.

In the field of multimodal data fusion, MLP emerges as a strong deep learning tool, renowned for its robust modeling capabilities and adeptness at autonomous feature extraction [31], particularly well-suited for handling high-dimensional and heterogeneous data [60]. In the current study, we observed that both deep learning and radiomics models emerged as promising independent diagnostic tools, boasting respective AUC values of 0.844 and 0.944, respectively. Furthermore, the fusion model that combined radiomics and deep learning features yield a higher AUC of 0.952, proving the effectiveness of incorporating epidemiological questionnaire data to assist CT-based diagnosis. Our study presented a holistic diagnostic method that integrates deep learning, radiomics and epidemiological data, and empirically demonstrated the capacity of MLP in the context of COPD diagnosis. This exceptional performance could be attributed to MLP’s proficiency in capturing intricate and nonlinear data relationships [61], a critical advantage in the complex domain of COPD diagnosis. Additionally, the high dimensionality of our dataset resulting from the integration of various features might have provided the MLP model with an advantage in discerning subtle interactions that linear models like LASSO [62] may overlook. Early diagnosis plays a crucial role in improving patient prognosis in COPD management, and our strategy proves to be a useful tool in assisting healthcare providers to integrate epidemiological data such as demographics, lifestyles, and health status of the patients into their diagnostic frameworks, especially in scenarios where COPD is suspected but not definitively diagnosed.

Moreover, our model can be easily deployed to clinical settings. Following chest CT scans acquisition, our all-encompassing workflow, including preprocessing, deep learning feature extraction, and radiomic feature extraction, supplies clinicians with a reference AUC for diagnosing COPD, regardless of the availability of patients’ epidemiological data. While multi-modal data have been collected routinely in clinical practice, how to utilize these data has been elusive. Our approach greatly facilitated this process and provided an example as utilizing the full potential of health big data.

Due to technical constraint, we were only able to investigate the SHAP values of the included predictors in each modal separately. Since both deep learning and radiomics features were abstract and hard to understand, we only investigated the variable importance of the epidemiological questionnaire data. Here we identified age, smoking, and tea intake as the top significant contributors to the model’s performance. It should be noted that these SHAP values were conditional on deep learning and radiomics features. To certain extend, our findings on these variables aligned with prior research on COPD risk factors. For instance, the influence of aging on COPD development might be attributed to factors like cellular senescence and heightened basal levels of inflammation and oxidative stress [63]. Smoking is widely recognized as the primary risk factor for COPD [64], with potential mechanisms of its role in airway inflammation [65] and vascular endothelial cell apoptosis [66]. Additionally, existing studies have indicated that the green tea consumption was associated with a decreased likelihood of developing COPD [67], potentially due to the tea extracts like catechin, which can reduce lung tissue inflammation [68]. These insights provided a robust foundation for the improved performance observed in our comprehensive models.

The strengths of our model include its high accuracy and potential for early intervention, which can improve patient outcomes in clinical practice. Additionally, the present model integrates readily accessible variables derived directly from CT images, thereby streamlining the diagnostic procedure, and rendering it user-friendly for healthcare practitioners. This accessibility promotes early intervention and enhances the management of COPD.

Despite its strengths, there are some limitations. The use of data from a single center necessitates external validation for model generalizability and stability. Future multi-center studies are needed to confirm the robustness of our modeling strategy. Additionally, optimizing feature selection and hyperparameter tuning as well as improving model interpretability are ongoing challenges that warrant further investigation.

## Conclusion

We successfully developed and demonstrated a comprehensive COPD early detection model by integrating deep learning and radiomics features from CT imaging, along with epidemiological data from questionnaires. Our proposed model provided clinicians with novel AI tools for COPD diagnosis, and can improve the prediction accuracy by incorporating epidemiological data, shedding lights on utilizing multi-source epidemiological and clinical data in diagnosis of COPD. This modelling strategy holds significant promise for practical implementation in clinical settings and serves as a valuable tool for COPD research.

## Data Availability

No datasets were generated or analysed during the current study.

## Abbreviations

COPD: Chronic obstructive pulmonary disease
AUC: Area under the curve
AI: Artificial Intelligence
CT: Computed Tomography
CNNs: Convolutional neural networks
MLP: Multi-Layer Perceptron
FTND: Fagerstrom Test for Nicotine Dependence
MET: Metabolic equivalent
PSQI: Pittsburgh sleep quality index
CES-D-10: Center for Epidemiological Studies Depression-10
LoG: Laplacian of Gaussian
LBP: Local Binary Pattern
GLCM: Gray Level Cooccurrence Matrix
GLSZM: Gray Level Size Zone Matrix
GLRLM: Gray Level Run Length Matrix
GLDM: Gray Level Dependence Matrix
NGTDM: Neighboring Gray Tone Difference Matrix
VAE: Variational Autoencoder
ELBO: Evidence Lower Bound
ReLU: Rectified linear unit
Adam: Adaptive Moment Estimation
SGD: Stochastic Gradient Descent
ROC: Receiver operating characteristic curve
